# The Changing Landscape of Autoimmune Hemolytic Anemia

**DOI:** 10.3389/fimmu.2020.00946

**Published:** 2020-06-03

**Authors:** Wilma Barcellini, Bruno Fattizzo

**Affiliations:** ^1^UO Ematologia, Fondazione IRCCS Ca' Granda Ospedale Maggiore Policlinico, Milan, Italy; ^2^Università degli Studi di Milano, Milan, Italy

**Keywords:** warm autoimmune hemolytic anemia, cold agglutinin disease, bone marrow transplant, checkpoint inhibitors, complement inhibitors, target therapy

## Abstract

Autoimmune hemolytic anemia (AIHA) is a greatly heterogeneous disease due to autoantibodies directed against erythrocytes, with or without complement activation. The clinical picture ranges from mild/compensated to life-threatening anemia, depending on the antibody's thermal amplitude, isotype and ability to fix complement, as well as on bone marrow compensation. Since few years ago, steroids, immunesuppressants and splenectomy have been the mainstay of treatment. More recently, several target therapies are increasingly used in the clinical practice or are under development in clinical trials. This has led to the accumulation of refractory/relapsed cases that often represent a clinical challenge. Moreover, the availability of several drugs acting on the different pathophysiologic mechanisms of the disease pinpoints the need to harness therapy. In particular, it is advisable to define the best choice, sequence and/or combination of drugs during the different phases of the disease. In particular relapsed/refractory cases may resemble pre-myelodysplastic or bone marrow failure syndromes, suggesting a careful use of immunosuppressants, and vice versa advising bone marrow immunomodulating/stimulating agents. A peculiar setting is AIHA after autologous and allogeneic hematopoietic stem cell transplantation, which is increasingly reported. These cases are generally severe and refractory to standard therapy, and have high mortality. AIHAs may be primary/idiopathic or secondary to infections, autoimmune diseases, malignancies, particularly lymphoproliferative disorders, and drugs, further complicating their clinical picture and management. Regarding new drugs, the false positivity of the Coombs test (direct antiglobulin test, DAT) following daratumumab adds to the list of difficult diagnosis, together with the passenger lymphocyte syndrome after solid organ transplants. Diagnosis of DAT-negative AIHAs and evaluation of disease-related risk factors for relapse and mortality, notwithstanding improvement in diagnostic approach, are still an unmet need. Finally, AIHA is increasingly described following therapy of solid cancers with inhibitors of immune checkpoint molecules. On the whole, the double-edged sword of new pathogenetic insights and therapies has changed the landscape of AIHA, both providing enthusiastic knowledge and complicating the clinical management of this disease.

## Introduction

Autoimmune hemolytic anemia (AIHA) has always been considered the simplest and most scholastic example of antibody-mediated autoimmune disease. As a matter of fact, autoantibodies (Ab) directed against erythrocytes, with or without complement (C) activation, are the main pathogenic mechanism of the disease (1). Clinically, it has long been considered a trouble-free disease, easy to treat, and with low clinical impact, compared with malignant hematologic conditions. This approach is quite similar to that of immune thrombocytopenia, which has been defined the “hematology's Cosette from Les Misérables.” More recently, AIHA has been identified as a greatly heterogeneous disease, due to several immunological mechanisms involved beyond antibodies, complement and antibody-dependent cell-mediated cytotoxicity (ADCC). Accumulating evidence demonstrates reduced CD4+ T-regs, imbalance of T-helper 1/2 cytokines, increased activity of cytotoxic CD8+ T lymphocytes, natural killer cells, and activated macrophages. More importantly, attention has grown on the pivotal role of bone marrow compensation, and on bone marrow characteristics that may reveal dyserythropoiesis, fibrosis, and clonal lymphoproliferation (1–4). Previously, steroids, immunesuppressants, and splenectomy were the mainstay of AIHA treatment (5–8). More recently, several new targeted therapies are increasingly used in the clinical practice or under development in clinical trials (7, 9). Along with new therapeutic options for patients, this growing armamentarium has complicated the clinical management of AIHA and increased the number of relapsed/refractory cases. Therefore, harnessing treatment and defining a risk-adapted therapy is an emerging unmet need. A peculiar setting is AIHA after autologous and allogeneic hematopoietic stem cell transplantation (HSCT), as well as cases described during therapy with immune checkpoint inhibitors for solid cancers (10). Finally, AIHAs may be associated with several conditions (lymphoproliferative, autoimmune and infectious diseases, immunodeficiencies, solid tumors, transplants, and drugs) where the several immunologic mechanisms are unpredictably involved (7, 11). The recent availability of next generation sequencing has improved the diagnosis of the several associated conditions, but at the same time has extended the proportion of “secondary” vs. “primary” AIHAs (4, 12). All these new insights in the pathogenesis of the disease and treatment opportunities have undoubtedly changed the landscape of AIHA.

In this review we will describe new diagnostic tools, clinical characteristics and therapeutic options of AIHA, focusing on relapsed/refractory cases, secondary forms, and AIHAs associated with HSCT or therapy with immune checkpoint inhibitors (CPIs). Moreover, we will approach the identification of risk factors for the development, clinical severity, response to therapy, and outcome of AIHA in order to start the basis for a risk-adapted therapy.

## Clinical Characteristics and Classification of AIHA

The gold standard for the diagnosis of AIHA is the Coombs test or direct antiglobulin test (DAT) that enables the classification of the disease according to the isotype and thermal characteristics of the autoantibody. Warm AIHA (wAIHA), the most common type (60–70% of cases) is typically DAT positive for anti-IgG, or IgG plus C, while cold forms (cold agglutinin disease, CAD, 20–25%), are due to IgM, and the DAT is positive for C3d. Among cold AIHAs it is worth considering paroxysmal cold hemoglobinuria (PCH), usually observed in children and; this very rare type of AIHA (1–5% of cases) is caused by the Donath-Landsteiner autoantibody, a bithermic hemolysin able to fix complement at cold temperatures and to determine RBCs lysis at 37°C. Mixed forms show both characteristics of wAIHA and CAD, with a DAT positive for both IgG and C and high titer cold agglutinins. Finally, there is a heterogeneous group of atypical AIHAs that include DAT negative, IgA driven, and warm IgM types (7, 8, 13). All these forms have a variable degree of anemia, hemolysis and bone marrow compensation, as shown in Table 1 for Hb and LDH levels, and reticulocyte counts.

**Table 1:** Clinical and laboratory characteristics of patients at onset divided according to AIHA serological type.

	**wAIHA (*n* = 225) IgG (*n* = 158); IgG +C (*n* = 67)**	**CAD (*n* = 107)**	**Mixed AIHA (*n* = 24)**	**Atypical AIHA (*n* = 22)**
**Hematologic features of primary AIHA patients**				
Median Age at diagnosis (years, range)	67 (5–94); 65 (21–92)	70 (28–94)	61 (20-86)	45 (25-78)
Hb (g/dL), median (range)	7.3 (2.1–14.1); 6.5 (2.0–11.5)	8.2 (4.0–13.5)	6.4 (4.3–10.7)	6.6 (3.0–10.9)
LDH (ULN), median (range)	1.7 (0.6–26.7); 1.8 (0.8–7.2)	1.4 (0.3–12.2)	1.7 (0.6–9.8)	2 (0.7–18.1)
Ret (×10^9^/L), median (range)	180 (22–644); 143 (53–641)	123 (13–644)	181 (45–576)	195 (29–780)
Inadequate reticulocytosis, *n* of pts (%)	86 (54); 35 (52)	69 (64)	15 (62)	14 (64)
**Hazard risks for AIHA relapse**				
Hb at onset	<6 g/dl	HR 1.98	95% CI 1.2–3.2	
AIHA type	Non wAIHA	HR 1.21	95% CI 0.9–1.5	
Evans Syndrome	Co-presence of ITP	HR 1.84	95% CI 1.2–2.7	
**Hazard risks for AIHA related death**				
Evans Syndrome	Co-presence of ITP	HR 8	95% CI 2.5–26	
AIHA related complications	Acute renal failure	HR 6.3	95% CI 1.4–29	
Multi-treatment (>4 lines)	Infections	HR 4.8	95% CI 1.5–15	

*wAIHA, warm autoimmune hemolytic anemia; CAD, cold agglutinin disease; IgG, DAT positive for IgG; IgG + C, DAT positive IgG + C; LDH (ULN), LDH is expressed as folds of upper limit of normal*.

### Risk Factors for Relapse and Mortality

Given the great clinical heterogeneity of the various AIHA forms, an effort has been made to identify predictors of outcome, including complications, response to therapy and death. The severity of anemia at onset has been identified as the strongest predictor of relapse, with hazard ratios of 1.61, 1.74, and 1.98, for Hb levels of 8.1–10, 6.1–8, <6 g/dL, respectively (5, 8). Complement involvement and thermal characteristics of the autoantibody were also important, with warm IgG+C, mixed, CAD, and atypical forms more frequently needing second or further therapy lines. Moreover, the concomitant presence of immune thrombocytopenia (Evans syndrome) is associated with a higher risk of relapse and refractoriness to treatment. Overall, AIHAs other than warm forms, plus Evans syndrome and Hb <8 g/dL at onset had a 4-fold increased risk of multiple relapses (8). Moreover, bone marrow features impact on disease severity since the presence of reticular fibrosis, dyserithropoiesis, and hypercellularity correlated with shorter relapse-free survival and lower response rate to immunosuppressive therapies (3). Regarding fatal outcome, Hb <6 g/dL at onset, Evans' syndrome, multi-treatment, acute renal failure, and infections have been associated with 5-8 fold risk of increased mortality (8). A case series of 13 very severe relapsed/refractory primary AIHA reported a mortality of 57%, despite intensive treatment, including transfusions, steroid boli, intravenous immunoglobulins, rituximab, erythropoietin, and plasma-exchange (13). More recently, mortality was 30% in a series of 44 AIHA admitted to intensive care unit for severe anemia (14). It is worth remembering that about 15–20% of AIHAs display thrombotic events, including severe episodes (pulmonary embolism, stroke, cardiac infarction), which are generally proportional to active hemolysis (5, 7). Risk factors for these severe, although not fatal, complications are Hb levels <6 g/dL at onset, increased LDH levels, and previous splenectomy (8).

### Secondary AIHAs

Several conditions represent a risk factor for the development of AIHA, including lymphoproliferative and autoimmune diseases, immunodeficiencies, infections, and solid tumors (Table 2). Concerning lymphoproliferative disorders, CLL patients show the highest risk with up to 5–10% developing AIHA, with an onset that may precede the diagnosis of lymphoproliferative disease (11, 15). The presence of unmutated IGHV status, sterotyped IGHV frames, and unfavorable cytogenetics (chromosome 17p and/or 11q deletions) represent a risk factor for the development of AIHA (4, 15–17). Other recently identified risk factors were several down-regulated miRNAs, some of them known to be involved in autoimmune phenomena (4). Of note, a positive DAT without hemolysis is frequent in CLL. AIHA prevalence in NHL is 2–3%, with higher frequencies in some subtypes (13–19% in angioimmunoblastic T-cell lymphoma and 50% in marginal zone lymphoma) (7, 11). A particular setting is CAD, which is associated with an indolent clonal lymphoid infiltrate distinct from other NHL (2, 6). In this disease recurrent mutations of KMT2D and CARD11 have been identified in 69 and 31% of cases, respectively (18). Similar mutations have also been reported in Kabuki syndrome, a congenital disorder characterized by malformations, immune-deficiency, and development of autoimmune diseases (4, 18).

**Table 2:** Secondary conditions associated with autoimmune hemolytic anemia (AIHA).

	**Frequency**	**Results**
**Lymphoproliferative disorders**		
Chronic lymphoid leukemia and NHL	5–20%	Autoimmune cytopenias may frequently complicate chronic lymphoproliferative disorders and usually correlate with advanced disease and high biologic risk
KMT2D and CARD11	69 and 31% of cAIHA tested	Autoreactive B-cells display somatic mutations favoring proliferation
**Congenital syndromes and immunodeficiencies**		
Kabuki syndrome and Hemoglobinopathies	4–6%	AIHA and ITP are the most frequent autoimmune complications of Kabuki Syndrome; DAT positivity is frequent, but clinically overt AIHA is rarer in thalassemia (particularly beta intermedia, alloimmunized, and transfused pts)
ALPS; CVID; IgA deficiency	2–70%	AIHA is the most frequent autoimmune complication together with ITP and ES
Genes involved in PIDsTNFRSF6, CTLA4, STAT3, PIK3CD, CBL, ADAR1, LRBA, RAG1, and KRAS	40% of pediatric ES	Majority of pediatric ES display somatic mutations found in immunodeficiencies
**Autoimmune diseases**		
SLE, Systemic sclerosis; autoimmune thyroiditis; Sjogren Syndrome; IBDs; Autoimmune hepatitis/Primary biliary cirrhosis	1.4–14%	AIHA frequency is higher in pediatric than in adult patients with SLE. AIHA may be rarely associated to systemic sclerosis or Sjogren syndrome, Hashimoto thyroiditis and Graves' disease, ulcerative colitis, and autoimmune hepatitis.
**Genetic findings**		
HLA I and II	Case series	HLA-B8 and BW6 are strongly associated to wAIHA.
IGHV and IGKV region	>60% cAIHA	Specific IGVH and IGKV regions are related to AIHA development
TCRG and TCRB	50%	Pathogenic T-cells are clonally restricted in AIHA
CTLA-4 exon 1	73%	CTLA-4 signaling is defective in AIHA, particularly in CLL cases
Cytokine polymorphisms	41%	AIHA shows higher frequency of LT-α (+252) AG phenotype
**Infections**		
Parvovirus B19; HCV; HAV; HBV; HIVMycoplasma spp.; Tubercolosis; Babesiosis; Brucellosis; Syphilis; EBV; Respiratory Syncytial Virus	0.02–20%	ParvoB19 infection and HCV and its treatment correlate with AIHA development; case reports of association with AIHA are available for the other infectious agents.
**Drugs**		
Antibiotics (penicillins, cephalosporins, etc.), cytotoxic drugs (oxaliplatin, etc.), antidiabetics (metformin), anti-inflammatory drugs (diclofenac, etc.), neurologic drugs (α-methyldopa, L-dopa, chlorpromazine, etc.), cardiologic drugs (procainamide, etc.)	Case reports and reviews	Various mechanisms are demonstrated: hapten and drug absorption mechanisms; Immune/ternary complex mechanisms; autoantibody mechanism; non-immunologic protein formation; unknown mechanisms.
CLL therapy: fludarabine and Tyrosin kinase inhibitors	6–21%	Fludarabine induced AIHA may be avoided by rituximab association. Ibrutinib was associated to low risk of AIHA development in registrative trials in CLL
**Vaccines**		
Vaccines	0.8/100.000 person-years	AIHA was the rarest autoimmune complication in a population study
**Solid cancers**		
Thymoma;Ovarian/Prostate	1.29–30% autoimmune phenomena	Thymoma, prostate and ovarian carcinomas have the highest association with autoimmunity

*AIHA, autoimmune hemolytic anemia; wAIHA, warm; cAIHA, cold; ES, Evans syndrome; ITP, immune thrombocytopenia; DAT, direct antiglobulin test; CLL, chronic lymphocytic leukemia; ALPS, autoimmune lymphoproliferative syndrome; CVID, common variable immunodeficiency; SLE, Systemic lupus erythematosus; IBDs, inflammatory bowel syndromes*.

Regarding AIHA in the context of immune dysregulation, patients with systemic lupus erythematosus develop AIHA in 14% of pediatric cases and 3% of adults (7, 11). A close association has also been reported with thyroid autoimmune disorders, such as Hashimoto thyroiditis and Graves' disease. Several case reports exist for AIHA association with systemic sclerosis, Sjögren syndrome (SS), autoimmune liver disorders, and inflammatory bowel diseases (7, 11). Moreover, various immunodeficiencies have been identified as predisposing conditions for AIHA, including common variable immunedeficiency (19), IgA deficiency, and autoimmune lymphoproliferative syndromes (ALPS) (20). Interestingly, mutations in genes implicated in primary immunodeficiencies (*TNFRSF6, CTLA4, STAT3, PIK3CD, CBL, ADAR1, LRBA, RAG1, and KRAS*) have been detected in about half of pediatric patients with AIHA and ITP (Evans Syndrome, ES); mutated patients showed more severe disease with higher treatment requirement and fatal outcome (12). These findings underline the close link between autoimmunity and immunodeficiency, i.e., a shared condition of dysregulated immune system.

### Genetic Background and Exogenous Triggers for AIHA Development

Although not specifically involved in the changing landscape of AIHA, it is worth considering genetic factors and historically recognized exogenous triggers (4). Several old and recent studies demonstrated a strong association of AIHA with HLA-B locus, particularly HLA-B8 and BW6 (21), or a reduced frequency of the disease in subjects harboring the HLA-DQ6 locus (22) (Table 2). As regards humoral immune response, various variable regions of the immunoglobulin heavy and light chains (IGHV and IGKV) have been associated with AIHA, particularly IGHV4-34, IGHV3, and IGKV3-20 genes, responsible for I antigen binding, and mostly represented in CAD (6). Concerning cellular immunity, autoreactive clonal T- CD8+cells have been reported in about 50% of AIHA cases; moreover, polymorphism of the cytotoxic T-lymphocyte antigen-4 (CTLA-4) gene and of lymphotoxin-α (LT-α) may represent a risk factor for primary or secondary AIHA development (4).

Various infections have been associated with an increased incidence of AIHA, particularly Parvovirus B19 (associated with DAT positive hemolysis in up to 20% of cases and hepatotropic virus, mostly HCV and possibly related to interferon therapy (11). Moreover, cold agglutinin AIHA occurs in up to 3% of patients with infectious mononucleosis and Mycoplasma pneumoniae infection (7, 11). Finally, paroxysmal cold hemoglobinuria is almost invariably preceded by an infection, including syphilis and virus, particularly in children (7, 11). In addition there is a long list of drugs that have been proven or highly suspected to induce AIHA, including historical ones (α-methyldopa, procainamide, penicillins, cephalosporins, diclofenac, ibuprofen, thiazides, quinine, quinidine, metformin) and more recent molecules (cladribine, fludarabine, lenalidomide, oxaliplatin, teniposide, pentostatin) (7, 11). Concerning new small molecules (ibrutinib, venetoclax, and idelalisib) few case reports of treatment-emergent AIHAs have been published (4).

## Comprehensive diagnostic Approach and New Diagnostic Tools

**Figure 1:**
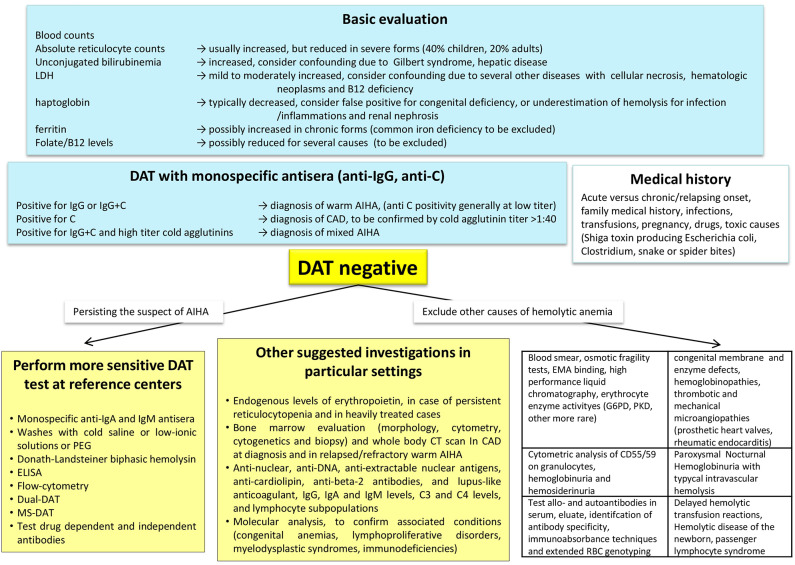
Diagnostic algorithm of autoimmune haemolytic anemia (AIHA). DAT, direct antiglobulin test or Coombs test; MS-DAT, mitogen-stimulated DAT; CAD, cold agglutinin disease; CT, computed tomography; G6PD, glucose-6-phosphate dehydrogenase deficiency; PKD, pyruvate kinase deficiency; EMA-binding, eosin-5′-maleimide-binding test.

Given the several associated conditions, an accurate diagnostic approach to AIHA is fundamental for a comprehensive risk assessment and a proper therapy (Figure 1). Medical history and baseline evaluation is still fundamental to assess drug assumption, infections, signs of acute or chronic hemolysis, and bone marrow compensation (reticulocytes).

### The Standard DAT and More Sensitive Techniques

As mentioned earlier, the DAT with monospecific antisera (anti-IgG, anti- IgA, anti-IgM, anti C) is the cornerstone of diagnosis, and allows a proper distinction of the various AIHA forms, that have different responses to therapy and prognosis (23). A diagnostic challenge that may take advantage of new diagnostic tools is represented by DAT-negative AIHA, usually 5–10% of all forms. In these cases, excluding other common causes of hemolysis and pursuing the clinical suspicion of AIHA, it is recommended to ask for second-level tests in a reference center. The DAT negativity due to low-affinity antibodies may be overcome by low ionic strength solutions (LISS) or cold washings. The small amounts of RBC-bound antibodies (below the threshold of the test) may take advantage of more sensitive techniques, such as microcolumn and solid-phase antiglobulin tests. In fact, DAT tube effectively diagnoses AIHA when at least 500 molecules of autoantibodies are bound to RBCs, whereas microcolumn and solid phase require ~200–300 molecules per single RBC to yield a positive result. Consistently, DAT tube is the most specific but least sensitive test, whereas microcolumn and solid phase methods show reduced specificity but increased sensitivity (7, 24, 25). Smaller amounts of autoantibodies can be detected by new, even more sensitive techniques, such as flow cytometry (able to detect about 30–40 antibody molecules per RBC), the enzyme-linked and radiolabeled tests, or the mitogen-stimulated-DAT (able to amplify the autoimmune reaction in culture) (23, 24). Of particular importance is the identification of atypical AIHAs due to warm IgM that are potent activators of complement and often detach from the RBC during washing procedures, causing detrimental delay in diagnosis and therapy. In these cases the DDAT (Dual Direct Antiglobulin Test) may allow the diagnosis of these rare forms, usually severe and potentially lethal (26). In addition, as complement activation is recognized as negative prognostic factor, evaluation of baseline values of C3 and C4 fractions, would help completing the diagnostic workup. Notwithstanding extensive evaluation, a fraction of AIHA remains DAT-negative: in these cases the diagnosis is made after the exclusion of the many hemolytic disorders (congenital hemolytic anemias, paroxysmal nocturnal hemoglobinuria, thrombotic microangiopathies, mechanical and toxic noxae) and on the basis of an ex-adiuvantibus therapy with steroids.

### Causes of Falsely Positive DAT

The DAT may be positive due to the presence of alloantibodies in recently transfused patients, in delayed hemolytic transfusion reactions, and in the hemolytic disease of the newborn (23). The coexistence of auto- and alloantibodies has been reported in about 30% of AIHA patients, and their presence is often masked by autoantibodies, possibly causing severe hemolytic reactions in case of RBC transfusion. In complex cases the distinction between allo- and autoantibody is advisable by immunoabsorbance techniques and by extended RBC genotyping (7, 24). It is worth reminding that daratumumab, the anti-CD38 antibody for the treatment of multiple myeloma, may give false DAT positivity. CD38 is also expressed on red-cell membranes, resulting in panreactive agglutination in the test used for antibody screening and cross-matching. Several methods have been proposed to overcome this interference, including pretreatment of red cells with dithiothreitol, use of antiidiotypic antibodies against daratumumab, supplementation of soluble CD38 to bind daratumumab in patient serum, use of red cells from newborns as test cells, and use of F(ab′)2 fragments of daratumumab by digestion with pepsin (27).

### Bone Marrow Evaluation and Exclusion of Secondary AIHA Forms

Among “new” diagnostic approaches to AIHA there is the increasingly recommended (and performed) bone marrow evaluation (morphology, cytometry, cytogenetics and biopsy). Bone marrow evaluation may in fact give important information on adequate erythroid compensation, underlying lymphoproliferative disorder, and evidence of an early/subclinical or therapy-related myelodysplasia or bone marrow failure. These features may help in harnessing therapy, avoiding further detrimental immunesuppression, or selecting immunosupprors among the new targeted therapies, based on the type of bone marrow lymphocyte infiltrate (T or B). Moreover, the determination of endogenous EPO levels may indicate this treatment, which has recently shown effective particularly in relapsed/refractory and heavily treated subjects (7, 28). To properly identify secondary forms imaging and serologic investigation is fundamental (Figure 1). It is advised to test for anti-phospholipid antibodies (cardiolipin, beta-2, and lupus-like anticoagulant), given the known thrombotic diathesis of acute/severe AIHAs, and thus advising thromboprophylaxis. Finally, molecular analysis and next generation sequencing would help confirming associated conditions (primary immunodeficiencies, lymphoproliferative disorders, myelodysplastic syndromes, other coexisting congenital anemias) again harnessing therapy.

## New Treatments for AIHA

The availability of several new treatments has undoubtedly boosted the therapeutic possibilities for patients, but at the same time has increased the number of heavily treated, relapsed/refractory cases. The immune-mediated pathogenic mechanisms in AIHA are different and may differently act at various degrees during the various phases of the disease. Therefore, the challenge for the future will be the selection and timing of administration of the several drugs available or under development. Firstly, distinction between wAIHA and CAD is pivotal, as therapy in quite different: the former usually respond to steroids, whereas the latter requires high and unacceptable doses. Splenectomy, although progressively abandoned and moved to third or further lines, is still a valid option for wAIHA; on the contrary it is ineffective and contraindicated in CAD, where RBC destruction occurs mainly in the liver and lymphoid organs. Likewise, it is poorly effective and discouraged in AIHA secondary to immunodeficiencies, autoimmune diseases, and lymphoproliferative disorders. Several target therapies are now in the clinical use or under development in AIHA (Table 3) (7, 9). Rituximab is becoming the preferred second-line for wAIHA and is recommended as first line in CAD. In the former, low-doses may be equally effective as standard ones, whereas in CAD the standard schedule is more effective. The drug is successfully administered in primary and secondary cases, alone or associated with chemotherapy (bendamustine, fludarabine, or other) in AIHAs secondary to lymphoproliferative diseases (6, 29, 30). The clinical challenges are wAIHAs relapsed after rituximab and unfit/refusing splenectomy, and CAD relapsed after rituximab monotherapy and unfit for rituximab-combined chemotherapy. In this setting, treatment selection would be ideally driven by disease-related risk factors, and/or associated conditions, as well as patient general comorbidities. Among monoclonal antibodies, ofatumumab (anti-CD20), alemtuzumab (anti-CD52), and daratumumab (anti-CD38) have shown promising results in case reports, mainly secondary AIHAs. Likewise, the orally administered B-cell receptor inhibitors ibrutinib and venetoclax seem particularly effective in secondary AIHAs, and the proteasome inhibitor bortezomib in 1/3 of refractory CADs (9). A further B-cell receptor target therapy is the PI3K inhibitor parsaclisib, which is under investigation in both wAIHA and CAD with very promising results. An interesting new approach for CAD is blocking complement activation, either at the C5 level (eculizumab) or more efficiently at the C3 (APL-2) or C1s level (sutimlimab), the latter with about 50% responses (31). Other drugs are directed at cellular immunity and cytokines, such as subcutaneous low-dose IL-2 and sirolimus (inhibitor of the serine treonine kinase mTOR). Targeting IgG driven extravascular hemolysis by inhibiting the spleen tyrosine kinase (Syk) is also an attractive approach in wAIHA (for example fostamatinib). Finally, inhibition of the neonatal crystallizable fragment receptor (FcRn) is a new interesting approach: these drugs avoid protection of circulating IgG, including pathogenic autoantibodies, from catabolism and thus regulate innate and adaptive responses initiated by IgG immune complexes (9).

**Table 3:** Target therapies in autoimmune hemolytic anemia (AIHA).

**Drug**	**Mechanism**	**Setting**	**Route of administration**	**Efficacy**
**B-cell directed monoclonal antibodies**				
Rituximab	Anti-CD20	wAIHA/CAD	IV	70–80%/50–60%
Rituximab	Anti-CD20	wAIHA/CAD	SC	100%
R-Fludarabine	Anti-CD20 + purine analog	CAD	IV	76%
R-CTX-Dex	Anti-CD20 + alkylator	WAIHA	IV	97%
R-Bendamustine	Anti -CD20 + alkylator	CAD	IV	71%
Ofatumumab	Anti-CD20	Secondary AIHA	IV	Case report
Alemtuzumab	Anti-CD52	Secondary AIHA	SC	Case reports
Daratumumab	Anti-CD38	Secondary AIHA	IV	Case reports
**B-cell receptor inhibitors**				
Ibrutinib	BTKi	Secondary AIHA	Oral	Case reports
Parsaclisib	PI3Ki	Primary wAIHA/CAD	Oral	Not available
Venetoclax	Bcl2	Secondary AIHA	Oral	Case reports
**Proteasome inhibitor**				
Bortezomib	Proteasome inhibitor	CAD/Secondary AIHA	IV	Case reports
Bortezomib	Proteasome inhibitor	CAD	IV	31.6%
**Complement inhibitors**				
Eculizumab	C5i	CAD/Mixed AIHA	IV	Case reports
Sutimlimab	Anti-C1s MoAb	CAD	IV	50%
APL-2	C3/C3bi	CAD/wAIHA	SC	50/40%
**T-cell directed therapies**				
Soluble IL-2	T-reg stimulation	wAIHA	SC	Not available
Sirolimus	mTORi	Evans'/Secondary AIHA	Oral	80%
Mycophenolate Mofetil	Purine synthesis inhibitor	wAIHA/CAD/Secondary AIHA/Evans'	Oral	81–100%
**IgG mediated phagocytosis inhibitors**				
Fostamatinib	Syki	wAIHA	Oral	44%
SYNT001	FcRn MoAb	wAIHA	IV	Not available
M281	FcRn MoAb	wAIHA	IV	Not available

*CTX, cyclophosphamide; BTKi, Bruton tyrosine kinase inhibitor; Bcl2, B-cell lymphoma 2; δPI3Ki, phosphatidylinositol-4,5-bisphosphate 3-kinase delta type inhibitor; MoAb, monoclonal antibody; mTORi, mammalian Target Of Rapamycin inhibitor; Syki, Spleen tyrosine kinase; FcRn, neonatal crystallizable fragment receptor*.

It is worth commenting that several trials with these new drugs are ongoing or being planned. In order to achieve meaningful endpoints it will be essential to properly select patients, bringing into consideration the number of previous treatments and related complications, associated conditions, intrinsic AIHA-risk factors, and type and degree of the immunologic dysregulation. This would provide the basis of a risk-adapted therapy in AIHA, as it is now advised for malignant hematologic conditions.

## The Transition From Chronic/Relapsing AIHA to Idiopathic Cytopenias/Dysplasias of Uncertain Significance (ICUS/IDUS)

**Figure 2:**
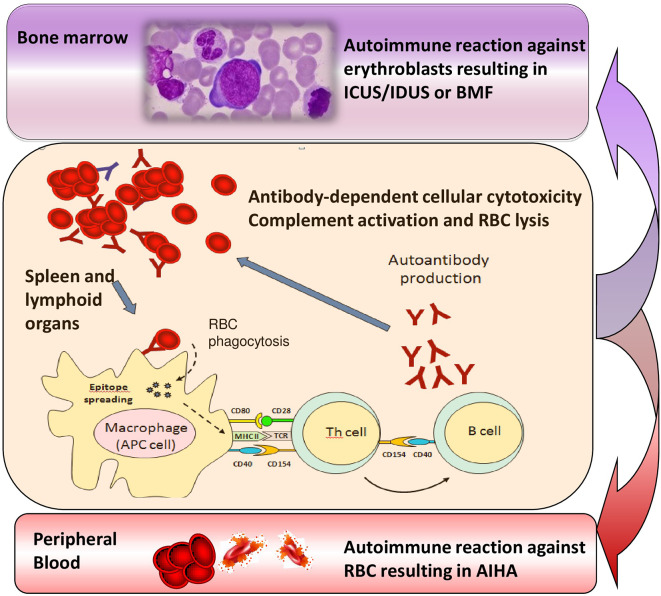
Transition from chronic/relapsing autoimmune hemolytic anemia (AIHA) to idiopathic cytopenias/dysplasias of uncertain significance (ICUS/IDUS) and bone marrow failure (BMF). Various immune effectors such as macrophages, antigen presenting cells (APC), T helper cells and B lymphocytes are involved into the immune attack, which is firstly directed against peripheral erythrocytes, but may persist and involve bone marrow precursors, possibly leading to ICUS/IDUS or BMF syndromes over time.

Several lines of evidence support the existence of a relationship between MDS and autoimmunity, including their epidemiologic association, the existence of common immune-mediated physiopathologic mechanisms, and the response to similar immunosuppressive therapies. This relationship may be hypothesized also with the recently-identified conditions ICUS and IDUS, which are defined by unexplained cytopenia (hemoglobin <10 g/dL; platelet count <100 × 10^9^/L; absolute neutrophil count <1.8 × 10^9^/L) and/or dysplasia in <10% of bone marrow lineages (32, 33). More recently another category has been proposed, the clonal cytopenias of undetermined significance (CCUS), where both unexplained cytopenias and clonal mutation are found, without fulfilling WHO criteria for MDS (34). Of note, about 10% of the general population aged over 70 years carries mutations in genes associated with myeloid neoplasms, usually single mutations at a low variant allele frequency, whose pathophysiologic role is still unknown. These cytopenias may be considered milder MDS forms that may evolve, after a variable period, in overt MDS or other bone marrow failure syndromes. In this view, we described the presence of anti-erythroblast antibodies in a case of erythroblastic synartesis, a rare disease with an autoimmune pathogenesis against erythroid precursors (35). Moreover, we also described two cases of AIHA and Evan's syndrome with anti-erythroblast antibodies, which showed a clear-cut bone marrow erythrocyte precursor hyperplasia at diagnosis, but evolved into IDUS and AA after several years (33). In this setting it is tempting to speculate that refractory/relapsing AIHAs lose their predominant “peripheral” pattern over time, and shift toward a “central” autoimmunity (Figure 2), leading to a refractory anemia. Additional factors, like accumulating somatic mutations, increased apoptosis, overinflammatory response (inflammaging), unfavorable bone marrow cytokine microenvironment, and breakdown of DNA-repairing tools (telomere shortening) are likely to play a role and need to be addressed in large prospective studies. The increasing availability of NGS panels will also help in defining the genetic background of the immunologic dysregulation, both in terms of inability to clear pathogens/external triggers (chronic infection), or failure to tolerate autoantiges (chronic autoimmune stimulation). This would be of great importance to selectively modulate (potentiate, down-regulate, or re-direct) the innate and adaptive immune response and to avoid an excessively toxic approach to autoimmunity.

## AIHAs Associated With Transplant

Organs and tissues transplants represent a challenging event for the recipient immune system and may evoke an “immunologic storm” resulting in either transplant rejection and/or devastating immune reactions. A particular mild picture is the passenger lymphocyte syndrome due to donor viable, immunocompetent lymphocytes present within the graft that can produce antibodies against donor RBCs. The syndrome involves mainly group O donors, though few cases have been described in AB recipients with non-AB donors. The risk of hemolysis is proportional to the burden of transplanted lymphocytes and ranges from 9 to 70% (kidney < liver < heart-lung transplants) (11, 36). Onset is between 3 and 24 days post-transplant and hemolysis is generally transient, since the lymphocytes transferred with the donor organ do not engraft. An emerging and more severe clinical entity is AIHA after hematopoietic stem cells transplant (HSCT). In this setting, autoantibodies are produced by the donor immune system against antigens on erythrocytes produced by the graft itself, and the clinical picture is generally severe (10). Several factors are implicated: the disease itself, the conditioning therapy preceding transplant, the subsequent immunosuppressive treatments, and the occurrence of HSCT complications such as viral infections reactivation. Moreover, the unfavorable immunologic microenvironment may lead to graft failure (as observed in transplanted patients with aplastic anemia), and graft immunocompetence may in turn induce graft vs. host disease (GVHD), further complicating the clinical course. Data from the literature report that immune hemolysis may complicate up to 2–4% of HSCTs after a median of 3–10 months. Both warm and cold forms are described, the former developing between 6 and 18 months, vs. 2–8 months for the latter. Risk factors for AIHA post HSCT are summarized in Table 4 and include use of unrelated donor and HLA-mismatch, occurrence of GVHD, use of cord blood, age < 15 years, CMV reactivation, alemtuzumab use, and non-malignant condition pre-HSCT (10). Mortality may be quite high and increases with infections (37).

**Table 4:** Risk factors and therapies for post-allogenic hematopoietic stem cell transplant (allo-HSCT) AIHA.

	**Risk factor**	**Estimated risk**	**95% confidence interval**	***P*-value**
**Risk factors associated with AIHA development post-allo-HSCT**				
Recipient	Age < 15 years	n.a.	n.a.	0.005
Disease features	Nonmalignant diagnosis pre-HSCT	3.5 (Hazard risk)^*^	1.1–10.9	0.031
Donor	Unrelated donor	1.45 (Relative risk)	1.05–1.99	0.02
	Unrelated donor	5.28 (Hazard risk)	1.22–22.9	0.026
	HLA mismatch donor	n.a.	n.a.	0.005
Source of stem cells	Cord blood use	n.a.	n.a.	0.005
Conditioning	Alemtuzumab use	2.5 (Hazard risk)^*^	1.1–5.7	0.028
Allo-HSCT complications	Chronic GVHD	12.17 (Relative risk)	96–1.54	0.018
	CMV reactivation	3.4 (Hazard risk)^*^	1.2–9.6	0.02
**Drug**	**Dose**	**N of patients**	**ORR (range)**	**N of line**
**Therapy of AIHA post-allo-HSCT**				
Wait & See	–	6	83%	–
Steroids	1–2 mg/Kg day	125	20% (10–50)	1st line
IVIG	2 g/Kg × 2 days	51	12% (10–50)	1st line
Splenectomy	–	18	38% (0–100)	2nd line
PEX	–	10	10 (0–14)	>2nd line
Rituximab	375 mg/sm/week × 4 weeks	18 125	89% (75–100%) 52% (36–100)	1st line 2nd line
Alemtuzumab	15 mg/day × 3/wk	2	50% (0–100)	>2nd line
Bortezomib	1,3 mg/mq	19	63% (25–100)	>2nd line
Sirolimus	3 mg/sm D1–1 mg/sm day	6	100%	>2nd line
Eculizumab	900 mg	3	33% (0–50)	>2nd line
Daratumumab	16 mg/Kg/week	3	100%	>2nd line
Abatacept	10 mg/Kg day	3	100%	>2nd line

*n.a. not available*.

* *refers to all the autoimmune complications; PEX, plasma exchange*.

### Therapy of Post-Transplant AIHA

Table 4 recapitulates current and novel therapies that have been used in AIHA post-HSCT. It is evident that the total number of patients reported in the various studies is small, and case reports and series carry the bias of describing good outcomes only. However, first-line steroids seem to work less than in primary AIHA, being effective in about 20% of cases only. Moreover, frontline rituximab appears much more effective than in second line (89 vs. 52% responses), and most Authors suggest its early use, particularly in severe cases. Splenectomy is effective but its use is limited to selected cases given the high surgical, infectious, and thrombotic risk. Regarding novel targeted therapies, alemtuzumab, bortezomib, sirolimus, eculizumab, daratumumab, and abatacept have all been used in selected cases, as 3rd or further line, with heterogeneous outcomes. Finally, the passenger lymphocyte syndrome may occur also in this setting and is favored by: use of cyclosporine alone for GVHD prophylaxis, use of peripheral blood rather than bone marrow as source of the graft, use of reduced-intensity conditioning, use of a non-genotypically HLA-matched donor, and use of a female donor. Umbilical cord blood as source for stem cells appears protective. Careful transfusion procedures are warranted in transplanted patients, particularly in mismatched cases (10, 36, 38).

## AIHAs Associated With New Biological Anti-Cancer Therapies

A fascinating field is that of anti-tumor immunotherapy, based on the understanding that tumor cells activate immune checkpoints such as molecular programmed death receptor-1 (PD-1) and cytotoxic T lymphocyte-associated antigen 4 (CTLA-4) signaling pathways to inhibit T lymphocyte activation and thus escape from immune surveillance, known as “immune brake.” Checkpoint inhibitors (CPIs) reactivate T lymphocytes to recognize cancer cells by blocking CTLA-4 or PD-1, and are therefore effective in numerous types of cancer. However, immune-related adverse effects have also been reported (39, 40) and hematologic ones are rare but potentially fatal. Most of them are monolineage cytopenia, or bilineage cytopenia, whilst acquired hemophilia A, eosinophilia, large granular lymphocytosis, and hemophagocytic lymphohistiocytosis are rare (41). A meta-analysis of 9,324 patients indicated that the incidence of anemia, neutropenia, and thrombocytopenia was 9.8, 0.94, and 2.8%, respectively (41). AIHA is the most commonly reported hematologic adverse event, with many case reports of fulminant course (40). A recent revision of the database of the Food and Drug Administration revealed a total of 68 cases: men to women ratio was similar, and the underlying diseases were mainly melanoma (41%), non-small cell lung cancer (NSCLC, 26%), and others including kidney cancer, Hodgkin's lymphoma or skin cancers. The reported cases were mostly from North America (49%) and Europe (34%), with a few from Asia (10%) and Australia (7%). Forty-three cases developed after nivolumab, 13 with pembrolizumab, 7 with ipilimumab, and 5 with atezolizumab, and 16% of cases had received two CPIs. The median time to AIHA onset was 50 days, four patients had concurrent thrombocytopenia, other four endocrine abnormalities (thyroiditis, adrenal insufficiency or hypophysitis), and three gastrointestinal adverse events (colitis or hepatitis). Most cases were IgG positive warm AIHA, whilst CADs were rarer. All episodes were severe, with 80% of cases developing grade 3–4 transfusion-dependent anemia, and the risk appeared higher with PD-1 or PD-L1 targeting agents (0.15–0.25%) than with CTLA-4 inhibitors (0.06%). Mortality was as high as 17%, mainly due to multi-organ failure and delayed diagnosis (42). In another recent analysis of 14 cases who developed AIHA after CPIs, median time to AIHA was 55 days (IQR 22–110 days). Compared to primary AIHA, these cases showed a higher proportion of DAT negativity (38%) and of severe anemia (median Hb 6.3 g/dL (IQR, 6.1–8.0 g/dL). Finally, 50% of cases relapsed after first line and 14% became chronic (43). Regarding therapy, prednisone 1.5 mg−2 mg/kg per day along with CPIs discontinuation is recommended, and evidences for the need of early rituximab or further immunosuppressive agents are lacking. The rechallenge of CPIs after AIHA has improved or is stable remains inconclusive. A patient with Hodgkin's lymphoma who developed nivolumab associated AIHA, that recovered after steroids and was later re-challenged with nivolumab without AIHA recurrence, has been described (44).

## Conclusions

Nowadays the pathogenic and therapeutic landscape of AIHA is rapidly changing for several reasons. First, numerous AIHA-associated conditions have been identified, such as autoimmune diseases, immunodeficiencies, and tumors, which may have additional immune-mediated pathogenic mechanisms compared to primary disease, and deserve a specific therapeutic approach. In this view, the increasing use of molecular testing has disclosed several underlying conditions, questioning the distinction between primary and secondary forms. Second, the development of new drugs has offered additional therapeutic opportunities to “cure” the disease, but at the same time has increased the number of relapsed/refractory cases. Moreover, the future availability of even more target therapies will further puzzle the treatment algorithm of the disease. Third, there is increasing awareness of various pathogenic mechanisms that may differently act during the disease course, ranging from a predominant “peripheral” autoimmunity against erythrocytes to a “central” attack against erythroid precursors, possibly preceding a myelodysplastic or aplastic evolution. These findings have further therapeutic implications, suggesting to avoid heavy immunosuppression in favor of immunomodulating/stimulating agents. Finally, there is increasing emergence of complex and severe entities, particularly AIHA developing after HSCT and AIHA associated with novel anti-cancer drugs such as checkpoint inhibitors, which represent a clinical challenge for complications and fatal outcome. Diagnosis of DAT-negative AIHAs and evaluation of disease-related risk factors for relapse and mortality have improved, but are still an unmet need. The assessment of disease-related risk factor would be pivotal to design good clinical trials and to give hints for a risk-adapted therapy of AIHAs.

## Author Contributions

WB designed the study, searched the literature, analyzed data, wrote the manuscript, and participated in the final revision. BF searched the literature, analyzed data, wrote the manuscript, and participated in the final revision.

## Conflict of Interest

The authors declare that the research was conducted in the absence of any commercial or financial relationships that could be construed as a potential conflict of interest.
